# Neural activity in self-identified claustrophobic individuals under in-vivo stimuli: A human electroencephalography dataset

**DOI:** 10.1016/j.dib.2021.107733

**Published:** 2021-12-18

**Authors:** Dunya Moradi, Fariborz Rahimi, Reza Eyvazpour, Ali Jahan, Seyed Hossein Rasta, Mahdad Esmaeili

**Affiliations:** a Department of Biomedical Engineering, Faculty of Advanced Medical Sciences, Tabriz University of Medical Sciences, Tabriz, Iran; b Department of Electrical Engineering, Faculty of Engineering, University of Bonab, Bonab, Iran; c Department of Biomedical Engineering, School of Electrical Engineering, Iran University of Science and Technology, Tehran, Iran; d Department of Speech and Language Pathology, Faculty of Rehabilitation sciences, Tabriz University of Medical Sciences, Tabriz, Iran; e School of Biomedical Sciences, University of Aberdeen, Aberdeen, UK

**Keywords:** Electroencephalographic, EEG, Claustrophobia, in-vivo stimuli, Chamber

## Abstract

The electrocortical activity in claustrophobic situations is a very limited field of study and has recently caught researchers’ attention. This article represents a set of electroencephalographic (EEG) data obtained from twenty-two participants. The volunteers include 9 participants with self-identified claustrophobia and 13 healthy controls under in-vivo stimuli. The EEG data were recorded using Mitsar 31-channel EEG system. Before cortical signal recording, Individuals were asked to identify themselves as healthy controls or claustrophobic participants. The EEG data collection process consisted of three experimental conditions. In all conditions, the participants were asked to stay calm and keep their eyes open. The first experimental condition was at seated resting state in a relatively large and well-lit laboratory (8m × 15m) area. In the second experimental condition, the subjects entered a moderately-lit chamber and repeated the previous resting situation. The final condition of the EEG data acquisition was performed in the same chamber but with reduced dimensions. For each experimental condition, duration of data collection was approximately 300 s. This data can be used to understand the brain's response in claustrophobic situations through various statistical or data-driven methods.

**Specifications Table**

**Table:** 

Subject	Biomedical signal processing, Neuroscience
Specific subject area	Signal processing, Cognitive Neuroscience, Cognitive Psychology, Multichannel EEG acquisition
Type of data	Raw data of 31-channel EEG signals resting state and in-vivo stimuli
How data were acquired	Multichannel EEG data were acquired using a 31-channel EEG acquisition system from Mitsar(Mitsar Co. Ltd. Saint Petersburg, Russia). WinEEG Research Software was used to collect the data.
Data format	Raw (.txt) EEG data
Parameters for data collection	Number of channels: 31 Sampling frequency: 2000 Hz stored on-line at a sampling rate: 500 Hz Resolution: 24 bits per channel
Description of data collection	Two groups of participants (9 self-identified claustrophobic individuals and 13 healthy controls) were considered for the analysis. Three experimental conditions were considered: Resting with open eyes in: 1- open and quiet area, 2- moderately-lit, closed-door chamber, 3 – reduced-dimension, closed-door and moderately-lit chamber. In each experimental condition participants were asked to stay in a resting state and avoid any voluntary or unnecessary movement. For each experimental condition, duration of data collection was 300 s. Condition-2 was performed in a wooden chamber (180cm Length × 50cm Width × 90cm Height). Condition-3 was performed in the same wooden chamber with a reduced size (90cm Length × 50cm Width × 90cm Height).
Data source location	Faculty of Advanced Medical Sciences, Tabriz University of Medical Sciences, Tabriz, Iran
Data accessibility	Link: https://data.mendeley.com/datasets/hrgfwzs3sx/2 DOI: 10.17632/hrgfwzs3sx.2
Related research article	**Authors:** Dunya Moradi, Reza Eyvazpour, Fariborz Rahimi, Seyed Hossein Rasta, and Mahdad Esmaeili **Title:** Electroencephalographic Activity in Patients with Claustrophobia: A Pilot Study [1] **Published at:** Journal of Medical Signals & Sensors DOI: 10.4103/jmss.JMSS_62_20

## Value of the Data

• The dataset contains electroencephalographic signals that allow for analyzing the brain activity in self-identified claustrophobic participants.
• The dataset provides EEG data from both healthy controls and self-identified claustrophobic participants. This means opportunity for exploring and differentiating the cortical brain dynamics in the two groups in response to in-vivo stimuli.
• Is functional brain connectivity different in claustrophobic and Healthy populations? The data can help questions like this and will also help to extract the task-specific functional connectivity in this population.
• Possible different brain connectivity can affect cognitive performance in this group of individuals. It may be of interest for researchers and clinical experts to investigate possible discrepancies in cognitive processing through comparing outcome of their cognitive tasks to our data.
• The data is provided in a standardized format to allow for ease of access for multiple purposes.

## 1 Data Description

Recently, a research was published [2] on the study of claustrophobia from cortical activity point of view. However, this is a very limited field of study which might be due to practical difficulties in data collection from these individuals. Therefore, we have tried to make up for this shortage. This dataset presents electroencephalographic (EEG) data from self-identified claustrophobic and healthy participants under rest and in-vivo stimuli. Twenty-two participants’ EEG signals were recorded during resting and two experimental test conditions. Data acquisition was performed with a 31-channel Electro-Cap Mitsar (Mitsar Co. Ltd. Saint Petersburg, Russia) system based on the 10-20 international system. The channels whose data were collected include: Fp1, Fp2, F3, F4, F7, F8, FC3, FC4, FT7, FT8, C3, C4, T7, T8, CP3, CP4, TP7, TP8, P3, P4, P7, P8, O1, O2, FPz, Fz, FCz, Cz, CPz, Pz, and Oz. The reference potential was the average of electrodes A1 and A2 to which the other electrodes on the right and left hemispheres of the head were referred. The 'AFz' electrode is also connected to the ground potential. The raw data consisted of three approximately 300-seconds-long segments related to three conditions of resting and testing under in-vivo stimuli. Data were recorded at a sampling rate of 2000Hz, but were down-sampled and stored at a rate of 500 Hz.

**Fig 1:**
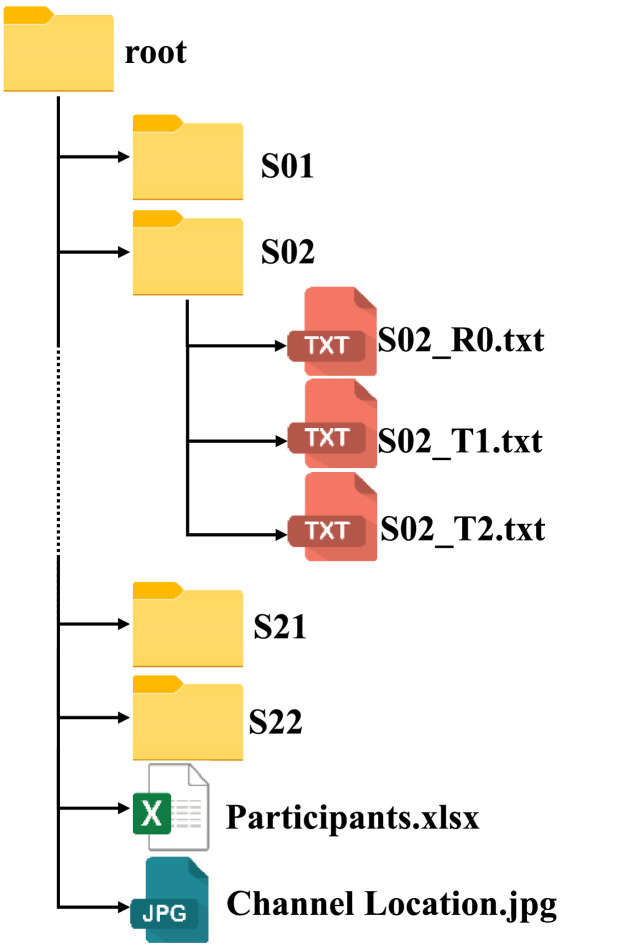
EEG dataset directory structure.

The entire dataset is hosted on Mendeley data repository (doi: 10.17632/hrgfwzs3sx.2) [3]. As shown in Fig. 1, the dataset consisted of 22 folders that represented the participants’ data, named S01 - S22. Each folder contains three .txt files that represent raw data. Raw EEG data is presented in a 31-channel format for each of the experimental conditions. In each .txt file, rows represent the 31 channels; and each column corresponds to data sampled at one time-stamp. Rest, Test-1, and Tests-2 conditions are represented by ``S[Participant Number]_R0 '', ``S[Participant Number]_T1'', and ``S[Participant Number]_T2'', respectively. A .jpg file presents detailed location of each channel's electrode, and a .xlsx file provides demographic data including participants' ID, age, gender, and self-reported labels for each participant.

## 2 Experimental Design, Materials and Methods

### 2.1 Participants

The EEG signals were recorded from 22 participants (9 with self-identified claustrophobia and 13 healthy controls). The age in claustrophobic group ranged from 23 to 48 years (mean ± std age = 29.11 ± 8.73, 44% female) and the age for healthy controls ranged from 21 to 47 (mean ± std age = 27.61 ± 6.39, 39% female). Demographic information of the participants is presented in Table 1. Participants were given an oral explanation of the experimental procedure involving their brain activity recording and provided an informed consent letter. It was explained that during the experiment, at any stage, if they feel uncomfortable, they can ask the procedure to be stopped immediately. For both groups, non-smokers, right-handed participants were included in the study with no history of neurological illnesses. They were asked to withheld taking any medication prior to the experiment session.

**Table 1.** The EEG signals recorded for each subject. Participants details.

Participant ID	Age (in years)	Gender	Claustrophobia/Non-Claustrophobia
S01	24	Male	Claustrophobia
S02	28	Male	Non-Claustrophobia
S03	25	Female	Claustrophobia
S04	23	Male	Non-Claustrophobia
S05	30	Male	Non-Claustrophobia
S06	24	Female	Non-Claustrophobia
S07	25	Male	Non-Claustrophobia
S08	25	Male	Non-Claustrophobia
S09	21	Female	Non-Claustrophobia
S10	25	Female	Non-Claustrophobia
S11	25	Male	Non-Claustrophobia
S12	25	Female	Claustrophobia
S13	27	Male	Claustrophobia
S14	48	Male	Claustrophobia
S15	25	Male	Claustrophobia
S16	39	Male	Claustrophobia
S17	47	Male	Non-Claustrophobia
S18	23	Female	Claustrophobia
S19	29	Female	Non-Claustrophobia
S20	29	Female	Non-Claustrophobia
S21	25	Female	Claustrophobia
S22	28	Male	Non-Claustrophobia

### 2.2 Experiment setup

**Fig 2:**
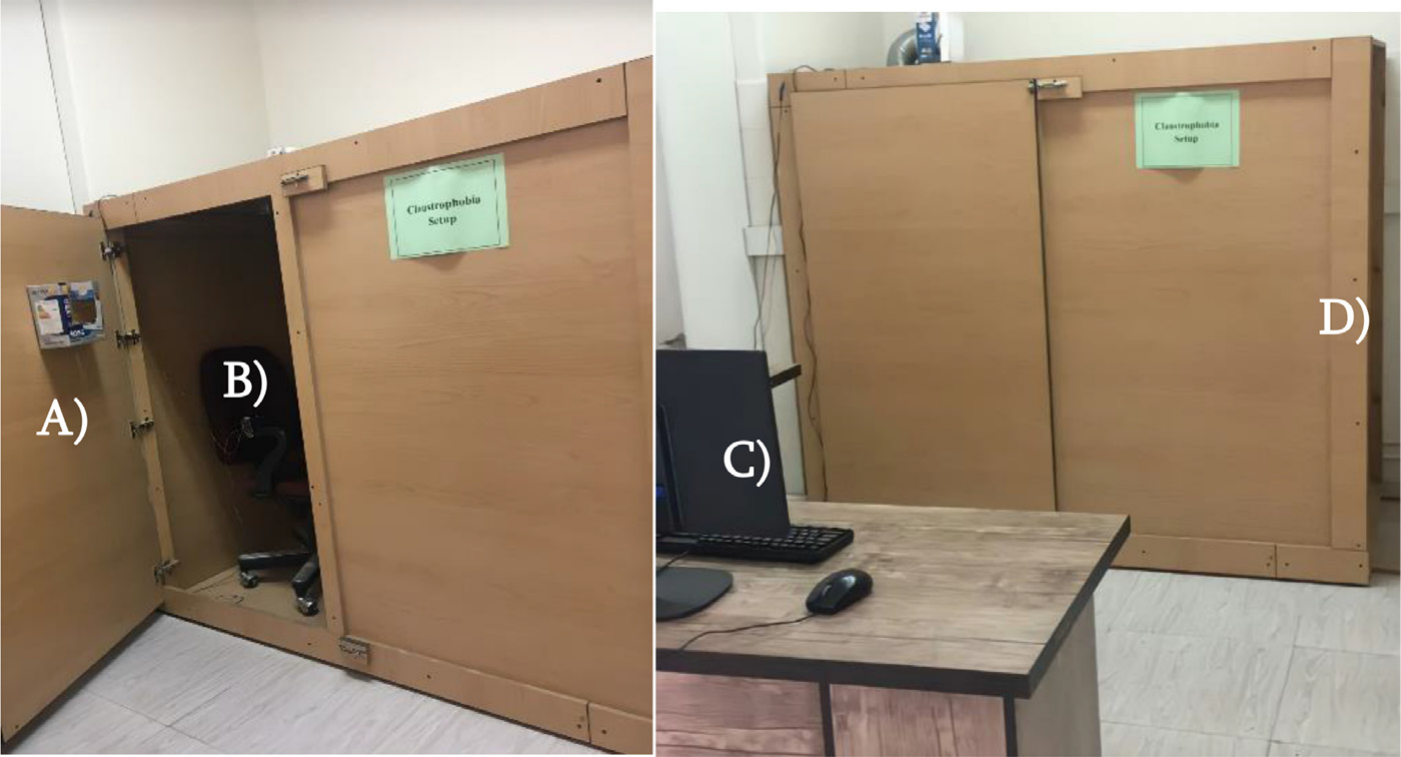
The experiment setup. A) Chamber door that is locked from outside. B) Participant chair for conditions 2-3. C) Data collection setup. D) Moveable wall to allow reduction of chamber space.

The experiment setup was a wooden chamber with dimensions of 90 cm × 180 cm × 155 cm (width × length × height). The various parts of the experimental setup are shown in Fig. 2. One side of this chamber was movable, which could reduce the dimensions of the chamber (to 90 cm × 110 cm × 155 cm). A comfortable chair outside the wooden chamber was placed in the laboratory for the resting state condition of experiment. For the second and third conditions of the experiment, the participant was asked to enter the wooden chamber and sit on a similar chair. Inside the wooden chamber, a ceiling air conditioning system was installed for proper air circulation to prevent the temperature inside the chamber from rising. Inside the chamber, proper lighting was provided using several small LEDs. Two metallic latch locks had been installed to prevent the door from opening from inside the chamber. Before the test, it was explained that the participant could request for immediate termination of experiment if they feel uncomfortable.

### 2.3 Experiment protocol

The experiment was performed in three consecutive conditions. The first condition was in a well-lit spacious laboratory. After setting up the data collection headset, the participant was asked to seat comfortably on a chair, for 300 s during which EEG data was recorded. In the second condition, the participant was asked to move into the experiment chamber and sit on a similar chair for 300 s for EEG data collection. In the third condition, the length of the wooden chamber was reduced to 110 cm (using the movable wall) and EEG data was recorded for 300 s. Participants were asked to stay relaxed and avoid stressful thinking during the whole experiment and a researcher at the computer monitor oversaw general activity throughout the experiment. The following paragraph explains the experiment procedure in detail.

A clear and detailed explanation of the experiment was given to each participant. Then, the guidelines to be followed during the experiment were provided (for example, avoiding sudden movements, talking or stressful thinking). To make experiment free from stress, enough time was given at any stage and if a participant felt not willing to continue (during or before each experimental condition), they could decide to cease the experiment, and the session would be terminated. Before the start of the experiment, materials and equipment were organized and participants were asked to sign a consent form. The recording device was ensured to be in good working condition and the participants were asked to make themselves comfortable in the sitting position. The EEG cap was placed over participant's head following the 10-20 standard positioning, and the device connection was checked for 100% connectivity. Each electrode was manually inspected for proper contact with the scalp before the start of the experiment. All the three consecutive testing conditions were recorded with the eyes open. In the rest condition, the chair was placed in a quiet and bright space and free of any claustrophobic stimulus. After the participant was placed in the desired position, and after placing the electro-cap, the signal was recorded.

Duration of data collection for all conditions were 300 s. Before the data collection in the second condition, when the participant had moved to the chair inside the wooden chamber, the impedance values of the electrodes were re-examined to ensure no increase in the impedance. The chamber door would be locked; and when the participant was ready data collection would be started.

## Ethics Statement

The voluntary, fully informed consent of the participants involved in this data collection was obtained on a written document. The study was conducted in accordance with the ethical standards laid down in the 1964 Helsinki Declaration and the ethical guidelines by the research ethics committee of Tabriz University of Medical Sciences (IR.TBZMED.REC.1398.690).

## CRediT Author Statement

**Fariborz Rahimi, Dunya Moradi** and **Ali Jahan:** Designed the study; **Dunya Moradi:** Collected the data; **Fariborz Rahimi, Reza Eyvazpour** and **Dunya Moradi:** Conceived the work and designed the article; **Fariborz Rahimi, Reza Eyvazpour** and **Dunya Moradi:** Wrote and revised the manuscript; **Fariborz Rahimi, Ali Jahan, Seyed Hossein Rasta** and **Mahdad Esmaeili:** Edited the manuscript; **Fariborz Rahimi:** Supervised the work. All authors (Dunya Moradi, Reza Eyvazpour, Fariborz Rahimi, Ali Jahan, Seyed Hossein Rasta, and Mahdad Esmaeili) agreed on the final manuscript before submission.

## Declaration of Competing Interest

The authors declare that they have no known competing financial interests or personal relationships which have or could be perceived to have influenced the work reported in this article.
